# Patent Foramen Ovale Closure for Treating Migraine: A Meta-Analysis

**DOI:** 10.1155/2022/6456272

**Published:** 2022-02-02

**Authors:** Yu Zhang, Haijiao Wang, Ling Liu

**Affiliations:** ^1^Department of Neurology, West China Hospital, Sichuan University, Wai Nan Guo Xue Lane 37, Chengdu 610041, Sichuan, China; ^2^Department of Neurology, Chengdu Shangjin Nanfu Hospital, Shang Jin Road 253, Chengdu 610000, Sichuan, China

## Abstract

**Background:**

Observational studies have shown percutaneous patent foramen ovale (PFO) closure to be a safe means of reducing the frequency and duration of migraine.

**Objective:**

This study evaluated the efficacy and safety of PFO closure in patients with migraine using evidence-based medicine.

**Methods:**

The Pubmed (MEDLINE), Embase, and Cochrane Library databases were searched for randomized controlled trials (RCTs), cohort studies, and retrospective case series from January 1, 2001, to February 30, 2021. The Jadad scale and R 4.1.0 software were used to assess the quality of the literature and meta-analysis, respectively.

**Results:**

In total, three randomized controlled trials, one pooled study, and eight retrospective case series including 1,165 participants were included in the meta-analysis. Compared with control intervention in migraine, PFO closure could significantly reduce headache frequency (OR = 1.5698, 95% CI: 1.0465–2.3548, *p*=0.0293) and monthly migraine attacks and monthly migraine days (OR = 0.2594, 95% CI: 0.0790–0.4398, *p*=0.0048). Subgroup analysis of patients who all completed PFO surgery showed resolution of migraine headache for migraines with aura (OR = 1.5856, 95% CI: 1.0665–2.3575, *p*=0.0227).

**Conclusions:**

Treatment with PFO closure could reduce the frequency of headaches and monthly migraine days and is an efficient treatment for migraine attacks with aura.

## 1. Introduction

Migraine is a common chronic neurovascular disorder characterized by self-limited, recurrent moderate-to-severe headaches associated with autonomic symptoms that affects 12% of the population [1, 2]. The patent foramen ovale (PFO) is present in 20–25% of the adult population but in 30–50% of those who have migraine with aura [3, 4]. Multiple studies have been conducted in the past showing that migraine, especially migraine with aura, is significantly related to the PFO [5–7]. Several studies have found that the incidence of PFO in migraine patients is 30–40% and is as high as 48–70% in migraine patients with aura (more than twice than that of the normal population) [4–7]. The pathogenesis of migraine in patients with PFA remains unclear. It may be a paradoxical embolism of venous microthrombosis [6]. Chemical substances, such as serotonin, are not cleared via pulmonary circulation, which triggers migraine. Multiple studies have reported improvement in migraine symptoms after transcatheter PFO closure [6]. The correlation between the PFO and migraine was originally reported in a case-control study conducted by Del Sette et al. in 1998 [8]. A meta-analysis conducted by Schwedt et al. in 2008 showed that the prevalence of PFO in patients with migraine ranged from 39.8 to 72%, and the prevalence of migraine in subjects with PFO also fluctuated between 22.3% and 64.3% [1]. To date, most single-center observations showed that PFO closure can effectively prevent migraine attacks, while three large randomized controlled trials (RCTs), MIST, PRIMA, and PREMIUM, have all reported negative results [9–11]. However, the latest study by Mohammad demonstrates that PFO closure was safe and significantly reduced the mean number of monthly migraine days and attacks, resulting in a greater number of subjects who experienced complete migraine cessation [12]. Therefore, the therapeutic effects of this surgical procedure remain controversial. Considering these inconsistent effects, we performed a systematic review and meta-analysis to revisit the utility and safety of PFO closure in migraine with and without aura.

## 2. Methods

### 2.1. Inclusion and Exclusion Criteria

The inclusion criteria were as follows: (a) type of study: RCT, cohort studies, and case-control studies; (b) language restrictions: English; (c) participating patients: patients with migraine; (d) intervention: PFO, placebo, or usual care; and (e) outcomes: resolution of migraine headache.

The exclusion criteria were as follows: (a) types of study: case reviews, case reports, meta-analysis, and reviews; (b) high rate of missed visits or follow-up time not in accordance with the study design; and (c) a study with the inability to extract OR values.

### 2.2. Information Sources and Search Strategy

Predesigned literature retrieval strategies were used according to the PRISMA guidelines [11], using “PFO closure,” “migraine,” and “patent foramen ovale closure” as search terms. Computer retrieval of relevant literature on treatment of migraine with patent foramen ovale closure was performed using databases such as the National Library of Medicine Biomedical Information Retrieval System (PubMed), the Dutch Medical Abstracts (EMBASE/SCOPUS), the Cochrane Library, and references of the included studies to supplement possible omissions of related clinical studies. The retrieval time was from January 1, 2001, to February 30, 2021.

### 2.3. Study Selection and Data Collection

Two reviewers independently evaluated the study records from the reference list and electronic database based on the aforementioned eligibility criteria. Differences were resolved through discussion or by a third evaluator. If data were missing from the literature, the authors were contacted as often as possible to obtain relevant information. After determining the studies to be included in the meta-analysis, two reviewers independently and in duplicate extracted information from each included trial according to our protocol. The extracted variables included the study type, sample size, age, and migraine headache resolution rate. Baseline data obtained after rigorous selection and assessment of the literature by the two reviewers are summarized in Table 1.

### 2.4. Quality Evaluation of the Literature

The Jadad scale was used to evaluate the quality of the literature [20]. (1) Method of generating a random grouping sequence: 2 points for using a computer-generated random grouping sequence or a random number table method; 1 point for a random assignment mentioned in the trial, but not given in the paper; and 0 points for a semirandomized or quasirandomized trial, which refers to the method of alternating allocation of cases, such as admission order, date of birth single, and double sign. (2) Randomization of concealment: 2 points for distribution schemes controlled by a medical center or pharmacy, use of numbered containers, present field computer control, using sealed opaque envelopes, or other methods that make it impossible for clinicians or subjects to predict the allocation of sequences; 1 point for only indicating the use of random digital tables or other random allocation schemes; and 0 points for alternating allocation, series numbers, series coded envelopes, or any measures that do not prevent grouping predictability or do not use randomization hiding. (3) Double-blind method: 2 points for describing the specific method used to implement the double-blind method and are considered appropriate, for example, a completely consistent placebo; 1 point for referring a double-blind method but are inappropriate methods in the literature; and 0 points for no reference to blind method. (4) Exit and missing visits: 1 point for mentioning and describing in detail the number and reasons of patients who withdrew and the number of cases lost to follow-up; 0 points for no mention of withdrawal or missing patients. With the highest possible score of 7, a score ≥4 indicated high quality and a score <4 indicated low quality. The high-quality clinical research included in this study was lower; therefore, it was included in the literature with a Jadad scale score ≥3.

### 2.5. Methodology for Statistical Analysis

All statistical analyses were performed using R 4.1.0. For continuous variables, we calculated the standardized mean difference (SMD) using the Mantel–Haenszel method in the risk factors for suicidal ideation, suicide attempts, and completed suicide in epilepsy. For counting variables, we calculated pooled odds ratios (ORs). The test level *α* of the effect was set to 0.05. Statistical heterogeneity was evaluated by the *I*^2^ statistic. The fixed-effects model was used for comparisons with *I*^2^ < 50%, and the random-effects model was applied for comparisons with *I*^2^ ≥ 50%. Sensitivity analysis was used to evaluate the stability of the meta-analysis results through the interconversion between the fixed-effects model and the random-effects model and to exchange statistical values to recalculate 95% CI, OR converted to risk ratio (RR), and SMD transformed to mean difference (MD). Egger's test was used to test the potential publication bias of the included literature, with *p* > 0.05 indicating the absence of publication bias [21, 22].

## 3. Results

**Figure 1:**
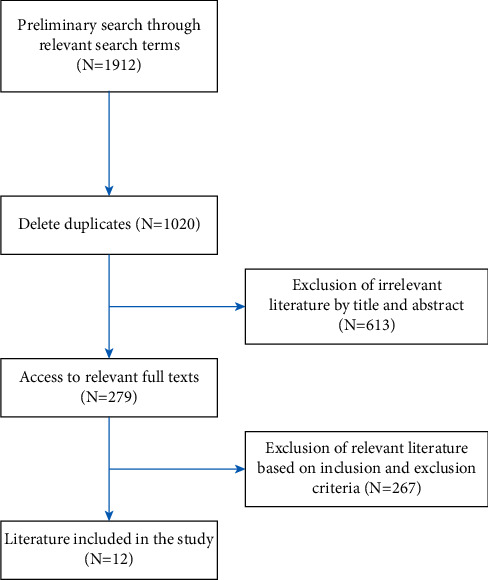
Flow chart of screening literature.

We identified 1912 titles and abstracts through PubMed, EMBASE, and Cochrane Library (Figure 1). After removing the duplicates and irrelevant articles, 1009 full-text articles were eligible for further evaluation. Again, after reading the titles, abstracts, and the full text, 891 articles were eliminated, and we were left with 12 articles [7, 9–24]. Eventually, three randomized controlled trials and eight retrospective case series with 1165 participants were included for quantitative synthesis. The main characteristics of the three randomized controlled trials, one pooled study, and eight retrospective case series are listed in Table 1.

### 3.1. Meta-Analysis Results

**Figure 2:**
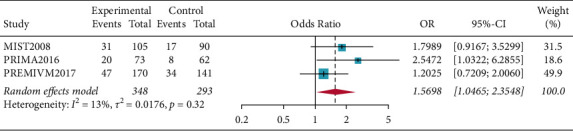
Forest plot for 50% reduction in monthly migraine attacks and monthly migraine days.

**Figure 3:**
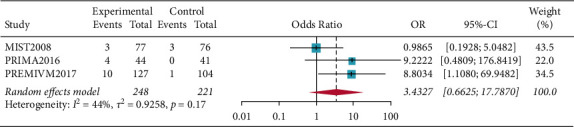
Forest plot for complete resolution of migraine headache.

**Figure 4:**
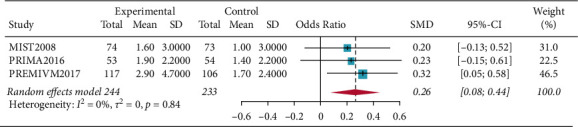
Forest plot for reduction in monthly migraine attacks and monthly migraine days.

**Figure 5:**
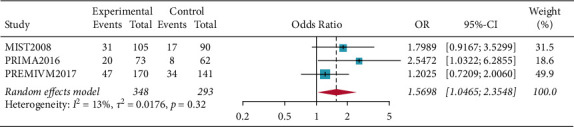
Forest plot for resolution of migraine headache for migraine with aura.

There were fifty percent reduction in monthly migraine attacks and monthly migraine days (OR = 1.5698, 95% CI: 1.0465–2.3548, *p*=0.0293) and reduction in monthly migraine attacks and monthly migraine days (OR = 0.2594, 95% CI: 0.0790–0.4398, *p*=0.0048). Subgroup analysis of patients who all completed PFO surgery showed resolution of migraine headache for migraines with aura (OR = 1.5856, 95% CI: 1.0665–2.3575, *p*=0.0227), and the results showed that patients in the PFO group experienced resolution of migraine headache. Complete resolution of migraine headache (OR = 3.4327, 95% CI: 0.6625–17.7870, *p*=0.1417) was not significant (Figures 2–5).

### 3.2. Safety and Adverse Events

Four studies included 484 patients with 28 serious adverse events in the PFO closure group [9–11], including 3 device-related events (transient atrial fibrillation, general fatigue, and syncope), 13 implant procedure-related events (access-site bleeding, retroperitoneal hematoma, arm phlebitis from an intravenous line, groin hematoma and pain, transient hypotension, tachycardia, and a vasovagal episode), and 12 unrelated events (muscle wasting, site bleeding, anemia, and nosebleed). All adverse events resolved without sequelae. During the follow-up of patients with a device, after at least 1 year, no device-related side effects were observed.

### 3.3. Sensitivity Analysis and Risk of Bias in Included Studies

**Figure 6:**
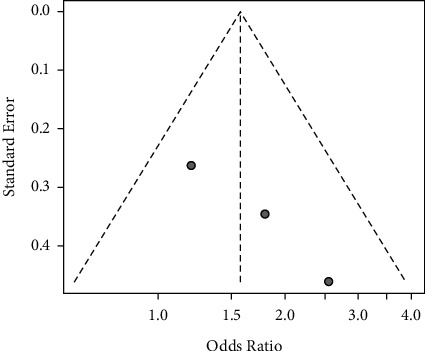
Funnel plots of the 3 RCT studies.

**Figure 7:**
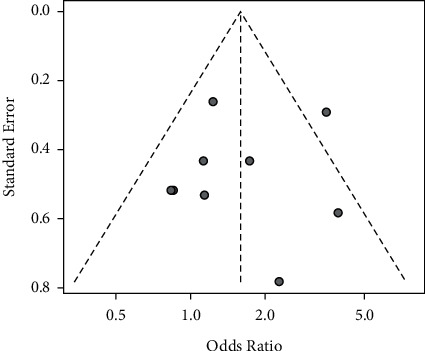
Funnel plots of the 8 RCS studies.

In the sensitivity analysis, we conducted a two-part sensitivity test on our results. The results were consistent, indicating that the results of the meta-analysis were stable (Table 2). The results of sensitivity analyses are shown in Table 2. There was no publication bias *p*=0.7108 (>0.05) in the included studies according to Egger's test, which means that the influence of publication bias on the results could be ignored (Figures 6 and 7).

## 4. Discussion

Migraine is a common disease in the world, affecting around 12% of the general population [6]. Despite various migraine-prevention interventions, migraines cause a significant burden to the affected patients. Estimates indicate that migraine is the sixth highest cause of years lost due to disability worldwide [19–25]. PFO has a close relationship with migraine, and previous studies have shown that treating PFO can reduce migraine pain [3–6]. At present, PFO occlusion is very mature. However, the research on the treatment of migraine with PFO occlusion is still limited, and the use of PFO occlusion to relieve migraine is still controversial [9, 10, 26]. Studies have shown that the migraine relief rate after PFO closure is as high as 50–80% [27]. In the meta-analysis of three to four randomized controlled clinical trials including 484 patients, we evaluated the effect of PFO closure on patients with migraine refractory to multiple medications. Our primary outcome of reduction in monthly migraine attacks and complete resolution of migraine headache was higher in the PFO closure group compared with that in the control group. Similarly, reduction in monthly migraine days was significantly better in the PFO closure group. This study found complete resolution of migraine headache not significantly, probably due to the small number of studies and the small number of complete headache relief.

Subgroup analysis of migraine patients who had performed PFO surgery found that patients with migraines with aura, in particular those with frequent aura, had a significantly greater reduction in migraine days and a higher incidence of complete migraine cessation following PFO closure. In patients with migraines without aura, PFO closure did not significantly reduce migraine days or improve complete headache cessation.

The presence of a precursor can be a predictor of improved migraine symptoms after PFO congestion. However, some studies have shown that some patients without aura do respond to PFO closure, which was statistically significant for the reduction of migraine attacks. However, in some patients, the frequency of migraine attacks increases within 4 weeks after the PFO closure, and the symptoms do not decrease until a few weeks later. It is speculated that the reason for this could be because the occluder activates the endothelial cells of the left heart, thereby activating platelets, which could be due to the increase in the concentration of serotonin in the vein. If serotonin is indeed the triggering substance for certain patients with migraines, then preventive dose antiplatelet therapy, such as aspirin and clopidogrel, can theoretically reduce migraine attacks.

The mechanisms by which PFO is involved in the occurrence of migraine include the following [21, 22, 28–31]: (1) The theory of abnormal thromboembolism: under normal circumstances, tiny venous blood clots or platelet aggregates are filtered through the pulmonary circulation. However, when PFO is present, these tiny emboli bypass the pulmonary circulation and directly enter the arteries, causing a short-term occlusion of the arteries, leading to hypoperfusion in the arterial blood supply area and triggering a migraine. This hypothesis can explain the phenomenon why antiplatelet drugs and anticoagulant drugs can reduce migraine attacks to a certain extent. However, some studies have found that the proportion of patients with visual aura and homocysteinemia in patients with both PFO and migraine is significantly higher, which may mean that not all patients with PFO have microembolisms. (2) Theory of vasoactive substances: vasoactive substances (5-hydroxytryptamine, calcitonin-derived gene-related peptide, etc.) can mediate the transmission of central pain signals and participate in the mechanism of migraines. Under normal circumstances, these vasoactive substances are inactivated by the monoamine oxidase in the pulmonary capillaries and do not enter the arterial blood. However, the PFO allows these vasoactive substances to bypass the lungs and directly escape to the systemic circulation, thereby entering the cerebral circulation in high concentrations and acting on the trigeminal ganglion cells, participating in the dural neurogenic inflammatory response, and thereby inducing a migraine. (3) Other mechanisms: some studies have found that an atrial shunt conforms to autosomal dominant inheritance, and the inheritance of migraine with aura in some families is similar to that of an atrial shunt. Studies have also found that the greater the degree of PFO shunt in patients with migraines, the more obvious the impairment of cerebral blood flow autoregulation. Therefore, the impaired dynamic cerebral blood flow regulation may play a role in the connection between PFO and migraine.

In the study of the treatment of migraine with PFO closure [9, 10, 26], six adverse events occurred in both the PREMIUM and PRIMA trials, including transient atrial fibrillation, syncope, hematoma, and phlebitis; 16 adverse events occurred in the MIST trial; and nine procedure-related adverse events and four device-related adverse events in the Mohammad trial. These may be related to the use of occluders in surgery. Although PFO occlusion may cause complications such as arrhythmia, phlebitis, retroperitoneal hemorrhage, aortic erosion, and occluder thrombosis, the incidence is low, and most of them are transient and recoverable complications and are routine after occlusion surgery. Administering antiplatelet drugs to prevent device-induced thrombosis can further reduce the risk of long-term stroke; hence, the occlusion is relatively safe.

The present study had several limitations. First, most of the included studies were retrospective, and there were only four randomized controlled trials, which might have limited the power of our analysis to measure significant differences in outcomes. Similarly, recall bias cannot be excluded. Second, the postsurgical therapy and the protocol for assessing the outcomes differed among the studies. Third, the surgical procedures used several different devices. Fourth, the abovementioned studies could be affected by the patient's recall deviation based on the degree of headache, the comfort effect brought by the operation, and the antiplatelet therapy drugs used in the perioperative period, which could also lead to a certain degree of bias. Finally, the baseline data on sex and age were not recorded in the four randomized controlled trials. Despite our attempts to contact the studies' authors, we could not obtain some data which would have enriched our analysis.

## 5. Conclusions

PFO closure was safe and significantly reduced the mean number of monthly migraine days and monthly migraine attacks, and the treatment was efficient for migraine attacks with aura. The results of this meta-analysis warrant a reevaluation of PFO closure in treating episodic migraine, especially for migraine with frequent aura.

## Figures and Tables

**Table 1:** Characteristics of included studies.

Author	Length of follow-up	Study type	Number		Safety of PFO closure	Jadad grade
			PFO group	Control group		
Schwerzman et al. [7]	1 Y	RCS	37	11	—	3
Azarbal et al. [13]	1 Y	RCS	24	13	—	3
Dubiel et al. [14]	Mean 38 M	RCS	24	22	—	3
Slavin et al. [15]	30 ± 16 M	RCS	41	10	—	3
Jesurum et al. [16]	1.5 Y	RCS	55	22	—	3
Mist et al. [9]	6 M	RCT	74	73	16 patients: pericardial effusion in 2 patients, 1 of which required percutaneous drainage, and a retroperitoneal bleed in 1 patient in the implant group, which was managed conservatively. Three serious adverse events that were probably related to antiplatelet medication (incision site bleed, anemia, and nosebleed)	6
Whal et al. [17]	5.0 ± 1.9	RCS	96	54	NO	3
Rigatelli et al. [18]	24–76 M	RCS	63	17	—	3
Prisma et al. [11]	1 Y	RCT	53	54	Six serious adverse events: three device related (one transient atrial fibrillation, one general fatigue, and one syncope), two related to the implant procedure (one access-site bleeding and one retroperitoneal haematoma), and one unrelated (muscle wasting)	7
Premivm et al. [10]	1 Y	RCT	123	107	Six major procedure-related adverse events: arm phlebitis from an intravenous line, groin hematoma and pain, transient hypotension, tachycardia, and a vasovagal episode	7
Eyal et al. [19]	1 Y	RCS	169	—	NO	4
Mohammad et al. [12]	10–12 M	RCT	157	146	Nine procedure-related adverse events (access-site hematoma and transient hypotension) and 4 device-related adverse events (paroxysmal atrial fibrillation)	7

RCT: randomized controlled trial; RCS: retrospective case series; M: months; Y: years.

**Table 2:** Sensitivity analysis of interconversion between the fixed-effects model and random-effects model and exchange of statistical values.

Risk factors	RR or MD 95% CI, *p* value	Exchange of statistic value RR or MD 95% CI, *p* value	Switching model OR or SMD 95% CI, *p* value
Reduction 50% in monthly (*N* = 3)	1.5698 [1.0465–2.3548], *p*=0.0293	1.3187 [1.1710–1.4867], *p* < 0.0001	1.7134 [1.2903–2.2602], *p*=0.0002
Complete resolution (*N* = 3)	3.4327 [0.6625–17.7870], *p*=0.1417	3.2345 [0.4567–12.5934], *p*=0.5623	3.0678 [0.7006–18.1714], *p*=0.3401
Monthly migraine days (*N* = 3)	0.2594 [0.0790–0.4398], *p*=0.0048	0.2531 [0.0789–0.4489], *p* < 0.0001	0.3024 [0.0678–0.4478], *p*=0.0001
Migraine with aura (*N* = 8)	1.5856 [1.0665–2.3575], *p*=0.0227	1.4356 [1.0345–2.6701], *p*=0.0002	1.6826 [1.0567–2.3456], *p*=0.0134

OR: odds ratio; RR: risk ratio; MD: mean difference; SMD: standardized mean difference; CI: confidence interval.

## Data Availability

The data used to support the findings of this study are included within the article.
